# Viral Myocarditis in the Setting of Delayed Manifestation of Hamman-Rich Syndrome

**DOI:** 10.7759/cureus.34916

**Published:** 2023-02-13

**Authors:** Ruchita Kabra, Sunil Kumar, Sourya Acharya, Pratik J Bhansali, Varun Daiya

**Affiliations:** 1 Department of Medicine, Jawaharlal Nehru Medical College, Datta Meghe Institute of Medical Sciences (Deemed to be University), Wardha, IND; 2 Department of Radiodiagnosis, Jawaharlal Nehru Medical College, Datta Meghe Institute of Medical Sciences (Deemed to be University), Wardha, IND

**Keywords:** interstitial, pulmonary, diagnosis, rapidly progressive, acute respiratory distress syndrome

## Abstract

Acute respiratory distress syndrome (ARDS)-like symptoms and rapid progression characterize the interstitial lung disease known as acute interstitial pneumonitis, also known as Hamman-Rich syndrome. It has a bad prognosis and a high incidence of mortality. We describe the case of a 25-year-old male patient with acute-onset type I respiratory failure with detrimental X-ray abnormalities who presented to the emergency room without any history of pulmonary disease or smoking. The provisional diagnosis of Hamman-Rich syndrome was reached after other clinical entities were ruled out based on CT findings. Myocardial hypokinesis of the apex and septum, as well as a modest systolic dysfunction (ejection fraction: 50%) similar to acute myocarditis, were detected by echocardiogram. Acute myocarditis in the setting of Hamman-Rich syndrome has been anecdotally reported and its mechanism remains to be elucidated.

## Introduction

The term "acute interstitial pneumonia" (AIP) refers to an idiopathic clinical and pathological disorder. Clinically, it can be differentiated from other, more enduring forms of interstitial pneumonia that develop quickly and result in respiratory failure. It is also known as Hamman-Rich syndrome and affects those who do not already have lung illness. The histopathological findings show diffuse alveolar injury. Hamman-Rich syndrome is characterized by the sudden onset of dyspnoea or tachypnoea with severe hypoxia or acute respiratory failure, as well as bilateral lung infiltrates on chest X-ray. Patients usually present with flu-like symptoms, which develop quickly. The etiology of Hamman-Rich syndrome is not well understood; however, it can result from a single trauma. This trait sets it apart from other similar disorders. Acute and chronic interstitial pneumonia differ in terms of pathologic lesions of varying ages and include interstitial inflammation in diverse lung locations, normal parenchyma, fibroblast foci, and honeycomb change [1]. Three phases of alveolar damage are associated with AIP: an initial exudative phase, a later organized proliferative phase, and a final fibrotic phase [2]. With a reported three-month mortality rate of 70% [3,4], the condition has a bad prognosis. The primary goal of treatment for this illness involves managing respiratory failure and its consequences. For the most part, the recommendations, which are based on a wealth of currently available evidence on the treatment of acute respiratory distress syndrome (ARDS), are directed toward concomitant respiratory dysfunction. Despite its ubiquity and the virus's poor prognosis, there is scarce information available on its cardiac consequences.

## Case presentation

**Figure 1:**
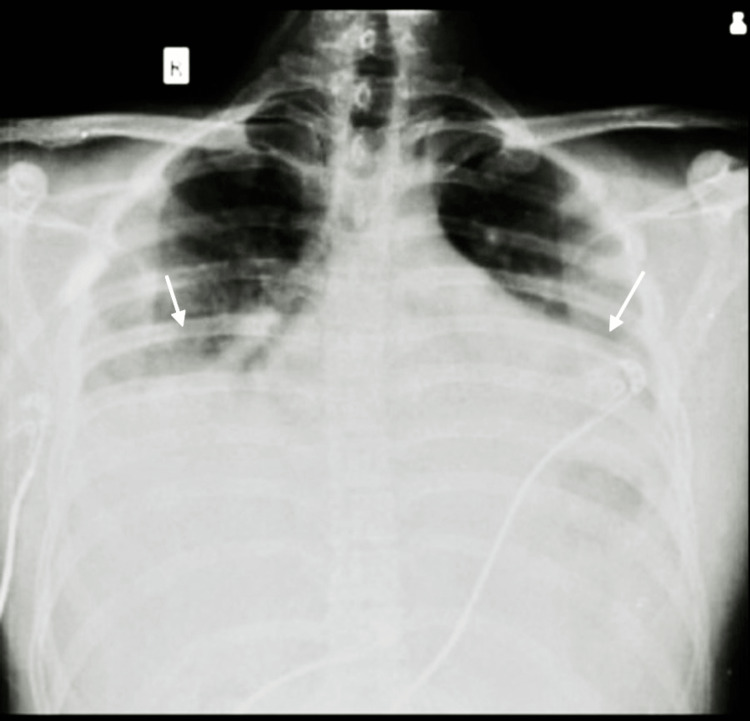
Chest X-ray showing bilateral opacities (arrows)

A 25-year-old male, a welder by occupation, presented to the hospital with a cough that had started suddenly and worsened progressively, eventually becoming productive and accompanied by shortness of breath. He had a history of fever for two days and loss of appetite for six to seven days, but no history of chest discomfort, palpitations, orthopnea, or paroxysmal nocturnal dyspnea during coughing. There was no previous history of similar episodes. The patient did not have any complaints of headaches, loss of consciousness, or seizures. He was admitted to the intensive care unit based on the above complaints and was extensively evaluated; the initial findings were as follows: a body temperature of 39 °C, pulse rate of 116 beats/minute, and blood pressure of 98/70 mmHg. On room air, oxygen saturation was 84%, while it was 96% on high-flow oxygen, with a respiratory rate of 38 beats/minute. The patient was kept on intermittent bilevel positive airway pressure (BiPAP) support due to tachycardia, tachypnoea, and dyspnoea. Chest radiography revealed opacities in the bilateral lower lung field (Figure 1).

His laboratory reports showed a normal white blood cell count of 10,300/cumm with predominant cells neutrophils, platelets count of 60,000/ul, high C-reactive protein (CRP) of 102.02 mg/L, aspartate aminotransferase (AST) of 84 u/L, alanine aminotransferase (ALT) of 98 u/L, alkaline phosphatase (ALP) of 195 u/L, erythrocyte sedimentation rate (ESR) of 110, and D-dimer of 581 mg/ml. To rule out infective etiology, antibodies for the malarial parasite, dengue, leptospira, and scrub typhus were done, which were negative.

**Figure 2:**
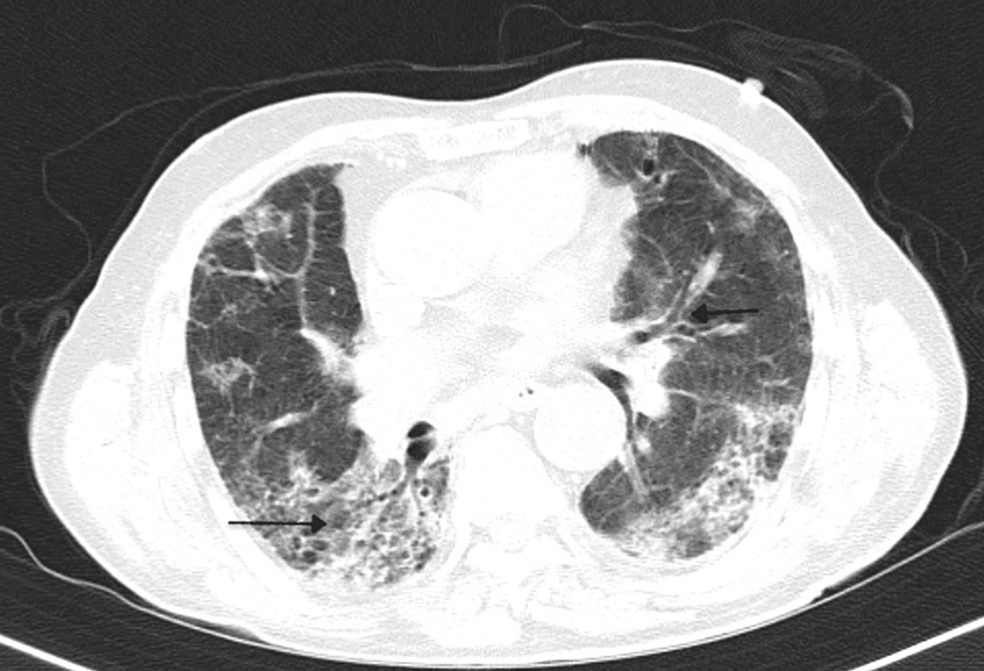
HRCT thorax image showing ground-glass opacities with patchy areas of consolidation (left arrow) and traction bronchiectasis (right arrow) HRCT: high-resolution computed tomography

His condition rapidly deteriorated on day two of admission with tightening of his chest and dyspnoea. His electrocardiogram suggested sinus tachycardia with cardiac enzymes CKMB-40 and negative troponin-I. Chest X-ray revealed diffuse bilateral opacities. Echocardiography showed standard valves, mildly dilated left ventricle, and mild global hypokinesia with pericardial effusion suggestive of myocarditis with an ejection fraction of 47%. CT of the thorax suggested multifocal ground-glass opacities with patchy areas of consolidation and tractional bronchiectasis (Figure 2).

A reverse transcription-polymerase chain reaction (RT-PCR) for coronavirus disease 2019 (COVID-19) was done, which was negative. Taking the above clinical evidence together with the patient's occupational history into account, he was diagnosed with AIP, aka Hamman-Rich Syndrome. He was treated with higher levels of intravenous antibiotics, steroids, diuretics, nebulization, and intermittent BiPAP with oxygen support. After six days of aggressive treatment, there was a subtle relief in his symptoms, and oxygen support was tapered and weaned off. The patient was shifted to the general ward for observation and was discharged after nine days of hospital stay. On follow-up, the patient was found to be doing well. His blood counts were repeated and were within normal limits.

## Discussion

Hamman-Rich syndrome is a clinicopathological condition described as sudden-onset widespread fibrosis of interstitial lungs, acute interstitial pneumonia, and an accelerated variant of interstitial pneumonitis. Current diagnostic criteria for AIP include idiopathic ARDS, clinical condition, and histological evidence of organized diffuse alveolar damage. It is also defined as the quick onset of respiratory failure in a previously healthy person who has never had a respiratory illness. The clinical presentation of AIP has been described in the literature [5]. The disease frequently strikes suddenly, with a prodromal sickness lasting one to two weeks before presentation. Cough, fever, and dyspnoea are the most prevalent signs [6]. It is not linked to cigarette smoking and affects men and women equally. The bulk of the patients is between the ages of 50 and 55 years [7]. While the exact cause of interstitial pneumonia is unknown, current research has indicated some plausible pathogenetic processes [8].

The diagnosis of AIP can be made clinically based on the idiopathic ARDS clinical presentation and the absence of other diagnoses after investigation. AIP diagnosis in many patients is made according to clinical history, physical examination, and noninvasive investigations. Biopsy of the lung should be avoided in these patients since it will not change their treatment plan [9]. A widespread, bilateral air-space opacification feature might be seen on a chest X-ray of the patients. Patchy ground-glass opacities with symmetrical and bilateral involvement are seen on the CT of the thorax. As a result, AIP is clinically and radiologically similar to ARDS. This AIP may be exacerbated by any viral infection, which must be ruled out [10,11]. In this case, viral markers for COVID-19 and H1N1 influenza were done, which were negative; however, in light of the persistent tachycardia, myocarditis was kept as one of the possibilities.

Supportive care, such as supplemental oxygen and ventilatory support, is usually the core aspect of therapy. Several studies have found that using glucocorticoids in treating AIP is beneficial, although others have reached the opposite conclusion [8]. Even with extensive therapy, such as mechanical ventilation, AIP has a high mortality rate (>60%), with the majority of the patients dying within six months of diagnosis [6]. For example, one who survives AIP has a near-zero recurrence rate and has complete recovery or near-total recovery of lung function [9]. In this case, we lost the patient to follow-up after two months. Hence, we could not perform a pulmonary function test.

Clinicians frequently use corticosteroids in conjunction with ventilation; however, their usefulness is debatable. Some studies have demonstrated that immunosuppressive therapy has a modest effect, while others have shown a beneficial effect and a better prognosis [12]. Even though lung-protective measures have been found to reduce mortality, AIP is still linked with a dismal prognosis. This sheds light on our limited understanding of the condition and, consequently, our inability to manage it effectively.

## Conclusions

In the event of an ARDS pattern, Hamman-Rich syndrome is frequently a diagnosis of exclusion. This case report illustrates the challenges clinicians face in correctly diagnosing and treating AIP. Early diagnosis may be hampered by the need to rule out any identified cause or predisposing factor. As an exclusionary diagnosis, a high index of suspicion is required to evaluate with clinical investigations, particularly in the early stages of the disease. AIP should be firmly kept as a differential diagnosis when encountering cases of quickly progressing interstitial pneumonia.
